# Molecular Determinants of GS-9620-Dependent TLR7 Activation

**DOI:** 10.1371/journal.pone.0146835

**Published:** 2016-01-19

**Authors:** Indrani Rebbapragada, Gabriel Birkus, Jason Perry, Weimei Xing, HyockJoo Kwon, Stefan Pflanz

**Affiliations:** 1 Department of Biology, Gilead Sciences Inc., Foster City, California, USA; 2 Department of Structural Chemistry Gilead Sciences Inc., Foster City, California, USA; Harvard Medical School, UNITED STATES

## Abstract

GS-9620 is an orally administered agonist of Toll-like receptor (TLR)7 currently being evaluated in clinical studies for the treatment of chronic HBV and HIV patients. GS-9620 has shown antiviral efficacy in preclinical models of chronic hepadnavirus infection in woodchuck as well as chimpanzee. However, the molecular determinants of GS-9620-dependent activation of TLR7 are not well defined. The studies presented here elucidate GS-9620 subcellular distribution and characterize its molecular interactions with human TLR7 using structure-guided mutational analysis. Based on our results we present a molecular model of TLR7 bound to GS-9620. We also determine that several coding SNPs had no effect on GS-9620-dependent TLR7 activation. In addition, our studies provide evidence that TLR7 exists in a ligand-independent oligomeric state and that, TLR7 activation by GS-9620 is likely associated with compound-induced conformational changes. Finally, we demonstrate that activation of NF-κB and Akt pathways in primary plasmacytoid dendritic cells occur as immediate downstream cellular responses to GS-9620 stimulation. The data presented here further our understanding of the molecular parameters governing TLR7 activation by GS-9620, and more generally by nucleos/tide-related ligands.

## Introduction

The Toll-like receptors (TLRs) are a family of pattern-recognition receptors that play a critical role in coordinating both innate and adaptive immune responses towards pathogens [1] [2] [3]. TLRs are type I trans-membrane proteins that contain several tandem leucine-rich repeat (LRR) motifs involved in ligand recognition and binding, a trans-membrane domain and a cytoplasmic Toll-IL-1 receptor homology (TIR) domain required for signal transduction [4]. TLR7, TLR8 and TLR9 constitute a subfamily of intracellular endo-lysosomal TLRs that recognize components of nucleic acids [5] [6] [7] [8]. TLR8 and TLR9 were shown to exist as preformed protein dimers, independent of ligand binding, which is in contrast to cell-surface TLRs where ligand binding induces receptor dimerization [9] [10] [11] [12]. Biochemical studies with TLR9 and crystal structures of TLR8 in complex with small molecule agonists indicate that ligand binding to preformed dimers induces a conformational change that juxtaposes two TIR domains, stabilizing a signaling-competent ‘active’ conformation [9] [10].

Although both TLR7 and TLR8 recognize components of single stranded-RNA (ss-RNA) including exogenous viral ssRNA and endogenous RNAs, they show distinct expression patterns [6, 13] [14] [15] [16]. TLR7 expression is limited to plasmacytoid dendritic cells (pDC) and B-cells, while TLR8 is predominantly expressed by monocytes, macrophages and myeloid dendritic cells (mDCs) [1] [17] [18]. Activation of TLR7 initiates the MyD88-dependent signaling cascade that results in the activation of several transcription factors, including nuclear factor κB (NF-κB) and interferon regulatory factors (IRFs) [19] [20] [13]. In pDCs, activation of TLR7 induces cell differentiation, increased expression of co-stimulatory molecules and type I IFN secretion [21] [22] [23]. TLR7 activation of B cells causes proliferation and increased Ig-secretion in both a cell intrinsic and IFN-α dependent manner [24, 25]. Thus, engagement of TLR7 leads to priming of adaptive immune responses and serves as a ‘danger signal’ to the host in response to extracellular microbial and viral ssRNA components.

GS-9620 is a small molecule agonist of TLR7 [26] [27] [28]. Orally administered GS-9620 has demonstrated promising antiviral efficacy in preclinical models of chronic hepadnavirus infection in woodchuck, as well as chimpanzee, and a recent Phase 1b clinical study showed a favorable safety profile of GS-9620 in chronic hepatitis B virus (HBV) patients [29] [30] [31].

Our studies sought to characterize the molecular determinants of GS-9620-mediated TLR7 interaction. Specifically, we investigated the sub-cellular distribution of GS-9620 and examined the most proximal biological events following TLR7 stimulation by GS-9620 in primary human pDCs. Through integration of structure-guided site-directed mutagenesis and a cell-based TLR7 reporter assay system we determined the molecular parameters required for GS-9620-induced stimulation. Our data highlight that TLR7 residues in LRR regions 11, 13, and 17 are critical for GS-9620-induced TLR7 signaling. Our data supports the previously reported structure model of GS-9620 binding to TLR7 [27] and provides new insight into the relative importance of various predicted interactions. Importantly, reported single-nucleotide polymorphisms (SNPs) in the coding region of TLR7 do not impact GS-9620 agonist activity. Additional results suggest that TLR7 can exist in a pre-formed ligand-independent, oligomeric state similar to TLR8 and TLR9. Overall, the data presented enhance our understanding of TLR7 activation by small molecule agonists, particularly GS-9620.

## Materials and Methods

### Cell culture, Antibodies and Reagents

Human embryonic kidney (HEK) 293 (CRL-1573, ATCC) and Huh-7 cells were cultivated in DMEM (Life Technologies) supplemented with 10% (v/v) heat-inactivated fetal bovine serum (FBS) (Hyclone Laboratories) and 1% (v/v) Penicillin-Streptomycin (Life Technologies) at 37°C in 5% CO_2_. Raw264.7a and Daudi (CCL-213, ATCC) cells were maintained in RPMI 1640 Glutamax (Life Technologies) with 10% (v/v) heat-inactivated FBS and 1% (v/v) Penicillin-Streptomycin at 37°C in 5% CO_2_. Human PBMCs were isolated from fresh whole blood derived from healthy human volunteers (AllCells) using standard Ficoll density gradient separation techniques. PBMCs were cultured in media containing RPMI 1640 Glutamax, 10% (v/v) heat-inactivated FBS and 1% (v/v) Penicillin-Streptomycin at 37°C in 5% CO_2_. Human pDCs were isolated from PBMCs obtained from healthy individuals using CD304 (BDCA-4) magnetic beads (Miltenyi Biotec) and maintained in complete media containing RPMI 1640 supplemented with 10% (v/v) heat-inactivated FBS and 1% (v/v) Penicillin-Streptomycin at 37°C in 5% CO_2_. Rabbit anti-human TLR7 polyclonal Ab (PA1-20817, Fisher Scientific), mouse anti-V5-Tag monoclonal Ab (R960-25, Life Technologies), mouse anti-HA-Tag monoclonal Ab (H3663, Sigma-Aldrich), anti-GAPDH monoclonal Ab (G8795, Sigma-Aldrich), HRP-conjugated secondary antibodies, goat anti-mouse (A4416, Sigma-Aldrich) and goat anti-rabbit (A6154, Sigma-Aldrich) were used. Anti Rab5 antibody was obtained from (ab18211, Abcam). Anti-human HLA-DR (641393), anti-CD123 (558714), anti-CD11c (561352), anti-NF-κB-PS529 (558422), anti-Akt pS473 (560858), and the Lineage cocktail 1 contained antibodies against CD3, CD14, CD16, CD19, CD20, CD56 (340546) were obtained from BD Biosciences. Bafilomycin A1 was purchased from Sigma-Aldrich. Resiquimod, CpG ClassA ODN 2216, IL-1β were purchased from (Invivogen). GS-9620 was synthesized by the department of medicinal chemistry, Gilead Sciences, Inc.

### Plasmids, Site-directed mutagenensis and Polymorphisms

Untagged pUNO-Human TLR7 constructs was obtained from Invivogen and used to generate C-terminal hTLR7-V5 tag and hTLR7-HA, hTLR7-GFP. Untagged human TLR9 was cloned into pUNO vector. NF-κB luciferase reporter pNiFty-Luc was obtained from Invivogen. TLR7 fragments containing point mutations and SNPs were generated by DNA 2.0 and subcloned into the original backbone vectors and verified by DNA sequencing. SNPs tested in this study were obtained from the National Center for Biotechnology Information dbSNP at http://www.ncbi.nlm.nih.gov/snp/.

### Sequence, homology modeling

A homology model of TLR7 was constructed, as previously described, based on an X-ray crystal structure of the human ectodomain (ECD) of TLR8 [27]. Briefly, the structure of the activated TLR8 dimer co-crystallized with Resiquimod (PDB code 3W3N) was used as a template for a model of human TLR7 [9]. The model was built in Prime 3.1, with a BLAST alignment and refinement of non-conserved residues (*Prime*, version 3.1; Schrödinger, LLC: New York, NY, 2012). The binding mode of GS-9620 in the resiquimod agonist pocket was investigated through conformational sampling in Macromodel 9.9 (*Macromodel*, version 9.9; Schrödinger, LLC: New York, NY, 2012). A model for human TLR9 was built in a similar fashion to support selection of appropriate point mutations.

### Intracellular accumulation of GS-9620

Daudi cells were incubated for indicated times with varying concentrations [^3^H] GS-9620 (0.7μCi/mL). Cell associated radioactivity was extracted with ice cold 80% ethanol and measured using liquid scintillation counting (Beckman-Coulter, Fullerton, CA). The total amount of GS-9620 in cells was calculated from a calibration curve for GS-9620 mass versus radioactivity. Cell volume was measured as previously described [32].

### Subcellular fractionation

Daudi cells (5x10^7^) were collected by centrifugation after one hour incubation with 30nM or 200nM [^3^H] GS-9620 (0.7μCi/mL). The cells were washed twice with an ice cold disintegration buffer containing 250mM sucrose, 25mM imidazol (pH 7.4), 5mM ATP and 1mM EDTA, and re-suspended in 500µL of the same buffer. Cellular membranes were lysed by passing the cells 15 times through 27G 1 ¼ needle, and nuclei were pelleted by centrifugation (2000g for 5 minutes). The suspension of organelles was laid on the top of 30% Percol in the disintegration buffer (6mL) and spun 32,000 rpm for 35 minutes in Beckman 50Ti rotor. Organelle fractions were collected from the top of Percol gradient and distribution of [^3^H] GS-9620 was measured using liquid scintillation counting (Beckman-Coulter). Lysosomal enzyme marker β-hexosaminidase, mitochondrial succinate dehydrogenase and ER maker NADPH cytochrome *c* reductase were assayed as previously described [33] [34] [35]. Early endosomes were detected by western blotting for Rab5 protein.

### Vesicular pH measurement

Raw264 cells were incubated overnight with 1mg/ml FITC-Dextran (Sigma-Aldrich FD70S). Cells were re-suspended in buffers containing 133 mM KCl, 30μM nigericin and 10mM Acetate, Phosphate or HEPES of varying pH (pH 4.0 to 7.6) or buffer containing various concentrations of bafilomycin for 10 minutes. An excitation scan from 400 to 515 nm was measured at emission wave length 530nm. Ratios of emissions at excitation wave lengths 495nm and 450nm were then plotted against pHs of buffers to generate a calibration curve. Change in pH at various concentrations of bafilomycin was calculated based on calibration curve. Mean values were generated and plotted using GraphPad prism software (Graphpad Software, LaJolla, CA).

### Cytokine assay

Plasmacytoid dendritic cells were enriched from human PBMC using “plasmacytoid dendritic cell isolation kit II” (Miltenyi Biotech), typically producing >80% purity of the pDC population. Then cells were seeded at 1000 cells per well and stimulated with GS-9620 or Resiquimod with or without 100nM bafilomycin A. IFN-α2 in medium was measured 48 hours post activation using ELISA kit (R&D Systems) as per manufacturer instructions. All assays were performed in triplicate wells. OD values were converted into pg/ml using standard curves generated as per kit specifications. Concentration of IFN-α2 was plotted using GraphPad Prism software.

### NF-κB reporter assay

5x10^4^ Huh7 cells were transfected with indicated plasmids together with reporter plasmid, pNifty- Luc (Invivogen) using Lipofectamine 2000 (Life Technologies). 24 hours post-transfection, cells were stimulated with GS-9620, Resiquimod, CpG ODN 2216, IL-1β or DMSO (vehicle). Four two-fold dilutions were performed for each of the above compounds tested starting at 5uM for GS-9620, at 10uM for Resiquimod, at 30uM for CpG ODN 2216, and at 12.5ng/ml for IL-1β. Cells were lysed after 20 hours stimulation and luciferase activity was detected using One-glo Luciferase Reagent (Promega). Luminescence was detected using the EnVision multilabel plate reader (Perkin Elmer). Luciferase activity was normalized to DMSO and reported as a fold-induction relative to the activity in DMSO treated cells. All assays were performed in triplicate wells. Mean values ± SEM were generated for each experiment and plotted using GraphPad prism software (Graphpad Software, La Jolla, CA).

### Immunoprecipitation

HEK293 cells transiently expressing human (h)TLR9 or wild-type hTLR7 or point mutations (hTLR7-F408A or hTLR7-D555A) that had C-terminal tag -V5 or HA, cultured in 10-cm dishes were stimulated as indicated for 24 hours. Cells were lysed using hypotonic lysis buffer, 50mM NaHPO4, 10mM NaCl, 0.01% n-Dodecly-B-D-maltoside (Sigma-Aldrich) with protease inhibitors UltraComplete (Roche). Lysates were clarified by centrifugation at 13,000 rpm for 15 mins. Cells lysates were incubated with Anti-V5-agarose beads (Life Technologies) for 3 hours at 4°C, washed 7 times with phosphate buffer saline with 0.01% n-Dodecly-B-D-maltoside. Washed beads were resuspended in Nupage loading buffer (Life Technologies) and boiled for 10mins. Samples were analyzed by SDS-PAGE under reducing conditions, followed by immunoblotting with anti-V5 or anti-HA mAbs.

### Immunoblotting

Samples were resolved on NuPAGE Novex 4–12% Bis-Tris SDS-PAGE gels (Life Technologies). Proteins were transferred to PVDF membranes (Life Technologies) using iBlot dry blotting systems (Life Technologies). Blots were blocked overnight at 4°C using 10% non-fat milk in phosphophate buffered saline with 0.05% Tween20. Membranes were probed with primary antibodies for 4 hours. Anti-human TLR7, anti-V5, anti-HA, anti-GAPDH and anti-Rab5 antibodies were used at 1:500, 1:5000, 1:2000, 1:2000 and 1:1000 respectively. Blots were washed 4 times and incubated with secondary antibody for 1 hour at 1:5000 for the goat anti-rabbit and goat anti-mouse-HRP conjugated antibodies. Blots were washed 4 times prior to be being developed using ECLPlus western blotting substrate (Pierce). Chemiluminescence was measured by ImageQuant LAS 500 (GE Healthcare Life Sciences).

### Phospho-flow cytometry

1x 10^6^ PBMCs from healthy donors (n = 6) were stimulated with GS-9620, Resiquimod or DMSO for indicated times and fixed with BD phosflo-Buffer I for 10mins at room-temperature and permed BD Perm Buffer III for 30mins on ice. Cells were stained using antibodies against p-NF-κB (pS529), p-Akt (pS473) and surface makers for 1 hour at room-temperature. Myeloid derived dendritic cells were defined as Lin^-^ (CD3, CD14, CD16, CD19, CD20, CD56) HLA-DR^+^ CD123^-^ CD11c^+^ while plasmacytoid dendritic cells were defined as Lin^-^ HLA-DR^+^ CD11c^-^ CD123^+^. Cells were acquired on BD LSR Fortessa X20 and analyzed using Flo-Jo software. Mean fluorescence intensity were normalized to DMSO treated cells for each time point. Analysis was performed employing three different approaches: (i) fold change MFI (which is the ratio of (absolute MFI observed with stimulating agent)/(absolute MFI observed with corresponding DMSO sample)); (ii) frequency of phospho-positive cells; (iii) by using a composite score of absolute MFI x frequency. Mean ± SEM of raw data was plotted using GraphPad prism software (Graphpad Software, LaJolla, CA).

### Statistical Analysis

Statistical analysis of results was performed where feasible or appropriate; statistical comparisons were performed using two-tailed, unpaired t-test.

### Expression and Purification of TLR7

Recombinant baculovirus encoding full length human TLR7 containing an N-terminal 6HIS tag was generated by amplifying the nucleotide sequence for residues 27–1049 of TLR7 by PCR. The PCR fragment was ligated into pFastBac1 (Invitrogen) which was modified to contain the honeybee mellitin signal sequence and a 6HIS tag.

TLR7 baculovirus was used to infect SF9 cells (Invitrogen). Cells were pelleted by centrifugation, flash frozen in liquid nitrogen, and stored at -80°C. The frozen cell pellet from 250ml cell culture was lysed using a dounce homogenizer. Cellular membranes were isolated by centrifugation (100,000 x g pellet), resuspended and TLR7 was solubilized with Fos-Choline 14 (Anatrace). The supernatant, containing solubilized TLR7 was purified by Ni-affinity, cation exchange, and size exclusion chromatography.

## Results and Discussion

### GS-9620 rapidly internalizes into cells and preferentially localizes to and signals from endo-lysosomal compartments

**Fig 1:**
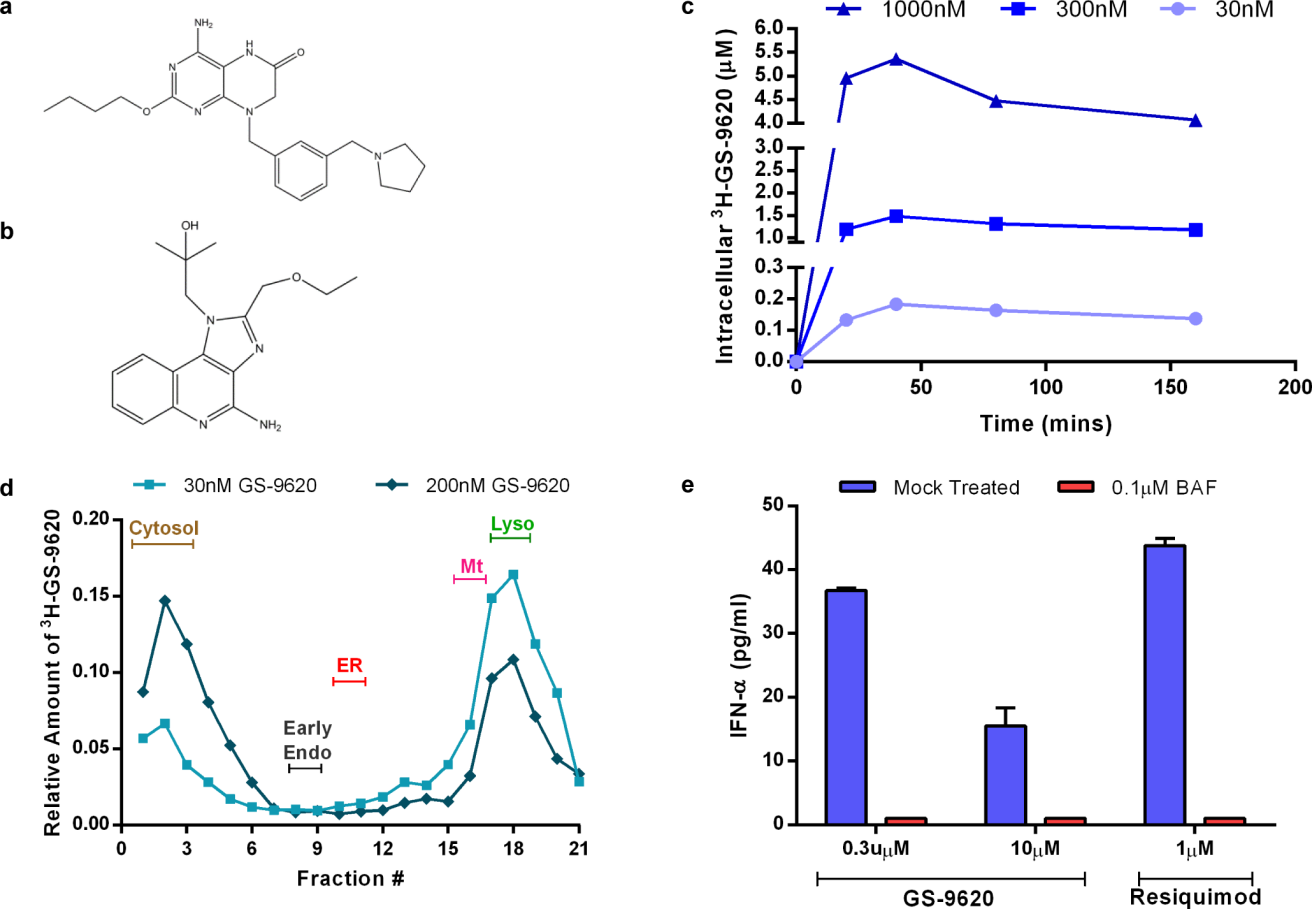
GS-9620 rapidly distributes to and signals through the endo-lysosomal compartments. (a-b) Chemical structure of (a) GS-9620 and (b) Resiquimod. (c) Kinetics of intracellular accumulation of ^3^H-GS-9620 in Daudi cells. (d) Sub-cellular distribution of ^3^H-GS-9620 in relation to major organelle markers obtained by isopycnic density gradient centrifugation. (e) IFN-α2 secretion by enriched pDCs upon stimulation with GS-9620 or resiquimod (control) in the presence of bafilomycin A1 (BAF) or PBS (mock). Data shown in (c), (d), (e) are representative of 3 independent experiments. (e) Error bars represent mean of triplicate assay conditions and SEM. Abbreviations: Early endosome (Early Endo), endoplasmic reticulum (ER), mitochondria (Mt), lysosome (Lyso).

Lipophilic molecules with partition coefficients greater than one (log*P*>1) and weakly basic amine moieties preferentially localize to acidic sub-cellular compartments such as late endosomes and lysosomes through a process known as pH partitioning [36]. Given that the log*P* value of GS-9620 was determined to be 3.3 and the pKa of the pyrrolidine group of GS-9620 was measured to be 8.0 (data not shown), we anticipated the preferential localization of GS-9620 in endo-lysosomes (Fig 1A). To test this hypothesis, we first measured the kinetics of cellular uptake of the compound in Daudi cells using tritiated GS-9620 (^3^H-GS-9620). The kinetics of ^3^H-GS-9620 accumulation was rapid, reaching concentration-dependent steady-state equilibrium in approximately thirty minutes (Fig 1C). Measured intracellular concentration of ^3^H-GS-9620 was 5-fold higher than the extracellular concentration of ^3^H-GS-9620 used to treat cells. Increases in intracellular ^3^H-GS-9620 concentrations were roughly proportional with increasing concentrations of ^3^H-GS-9620 (Fig 1C).

To assess the intracellular distribution of GS-9620 we performed sub-cellular fractionation studies with ^3^H-GS-9620 (S1 Fig). At lower concentrations (30nM), ^3^H-GS-9620 preferentially localized in fractions that contained lysosomes (Fig 1D). At higher concentrations (200nM), ^3^H-GS-9620 localized in both lysosomal and cytosolic fractions, possibly due to leakage of the compound from lysosomes during fractionation. Alternatively, this could also be due to saturation of GS-9620 within lysosomes or by another mechanism. Using the same approach, we determined that the dual TLR7 and TLR8 agonist resiquimod (Fig 1B) preferentially distributed into the cytosolic fractions while chloroquine localized to the lysosomal fraction (data not shown). Assuming lysosomes constitute approximately 0.5–5% of the total cellular volume, we estimate that at a 30nM GS-9620 extracellular concentration the lysosomal concentration would be approximately 2–20µM [37]. Taken together, the physiochemical properties of GS-9620 are likely responsible for the improved clinical potency and reduced off target interactions of GS-9620 relative to resiquimod [26] [27].

Previous, studies showed the importance of acidic endo-lysosomal pH in maintaining the stimulatory effects of TLR9 ligands [38]. Given the endo-lysosomal location of TLR7, we hypothesized that GS-9620 activity would also be affected by endo-lysososmal pH. Endo-lysosomal pH is maintained through the activity of vacuolar H^+^ ATPases (V-ATPase). Bafilomycin A1 is a specific inhibitor of V-ATPases that blocks endo-lysosomal acidification leading to increased pH within these compartments. GS-9620 was previously shown to induce secretion of type I IFN in PBMC cultures through the activation of pDCs [26]. The effect of bafilomycin A1 treatment on the induction of IFN-α by GS-9620 in pDCs was evaluated. pDCs isolated from blood of healthy human donors were either mock treated or bafilomycin A1-treated for 30 minutes prior to stimulation with GS-9620 or resiquimod. Secreted IFN-α2 was measured using ELISA in the supernatant after 48 hours. The amount of IFN-α secreted by pDCs induced with GS-9620 or resiquimod was 36pg/ml and 43pg/ml, respectively. Pretreatment with bafilomycin A1 reduced IFN-α2 induction by both GS-9620 and resiquimod to amounts below the level of detection (Fig 1E). Furthermore, 0.1μM bafilomycin A1 (the concentration used in the assay) increased lysosomal pH from 4.8 to 5.4 (S2 Fig). While GS-9620- or R848-induced secretion of IFN-α appears relatively low in this data set [21], he inhibition of GS-9620 induced IFN-α2 by bafilomycin A1 suggests that appropriate acidification of the endo-lysosomal compartment is critical for GS-9620 activity (Fig 1E). These results further support the concept that GS-9620 interacts with TLR7 within the endo-lysosomal compartment to induce IFN-α production in pDC.

### Structure based mutational analysis of TLR7 supports predicted binding pocket for GS-9620

We sought to define residues in human TLR7 that are critical for GS-9620 activity using an *in vitro* reporter assay system. Based on the crystal structure of TLR8/Resiquimod and homology models of TLR7/GS-9620 and TLR9, we performed structure guided mutational analysis of TLR7 [9] [27]. Expression constructs containing untagged, full-length wild-type TLR7, TLR9, or mutant TLR7 were cotransfected with an NF-κB-dependent luciferase reporter construct into Huh7 cells (constructs with C-terminal tags on TLR7 were inactive in our assay system, data not shown). Cells were stimulated with GS-9620 or control compounds and luciferase activity measured after 20 hours. GS-9620 dose dependently induced reporter activity in cells transfected with TLR7 (S3A and S3B Fig).

Several experiments were performed to develop and validate the experimental system. TLR7 transfected cells treated with 5μM GS-9620 induced an approximately 16-fold increase in luciferase activity as compared to DMSO (vehicle control) treatment. In contrast, GS-9620 did not induce luciferase reporter activity in cells transfected with TLR9, confirming selectivity of GS-9620 for TLR7 in this assay (S3A and S3B Fig). Additionally, GS-9620-dependent NF-κB reporter activation was confirmed to be sensitive to bafilomycin A1 treatment. Pretreatment of TLR7 transfected cells with bafilomycin A1 reduced GS-9620-induced reporter activity to baseline, indicating that TLR7 activation in our *in vitro* assay system indeed occurred in the endo-lysosomal compartment (S3C and S3D Fig). Using IL-1b stimulation as control we showed that MyD88-dependent signaling and reporter activity *per se* is not affected by bafilomycin A1 treatment (S3D Fig).

**Fig 2:**
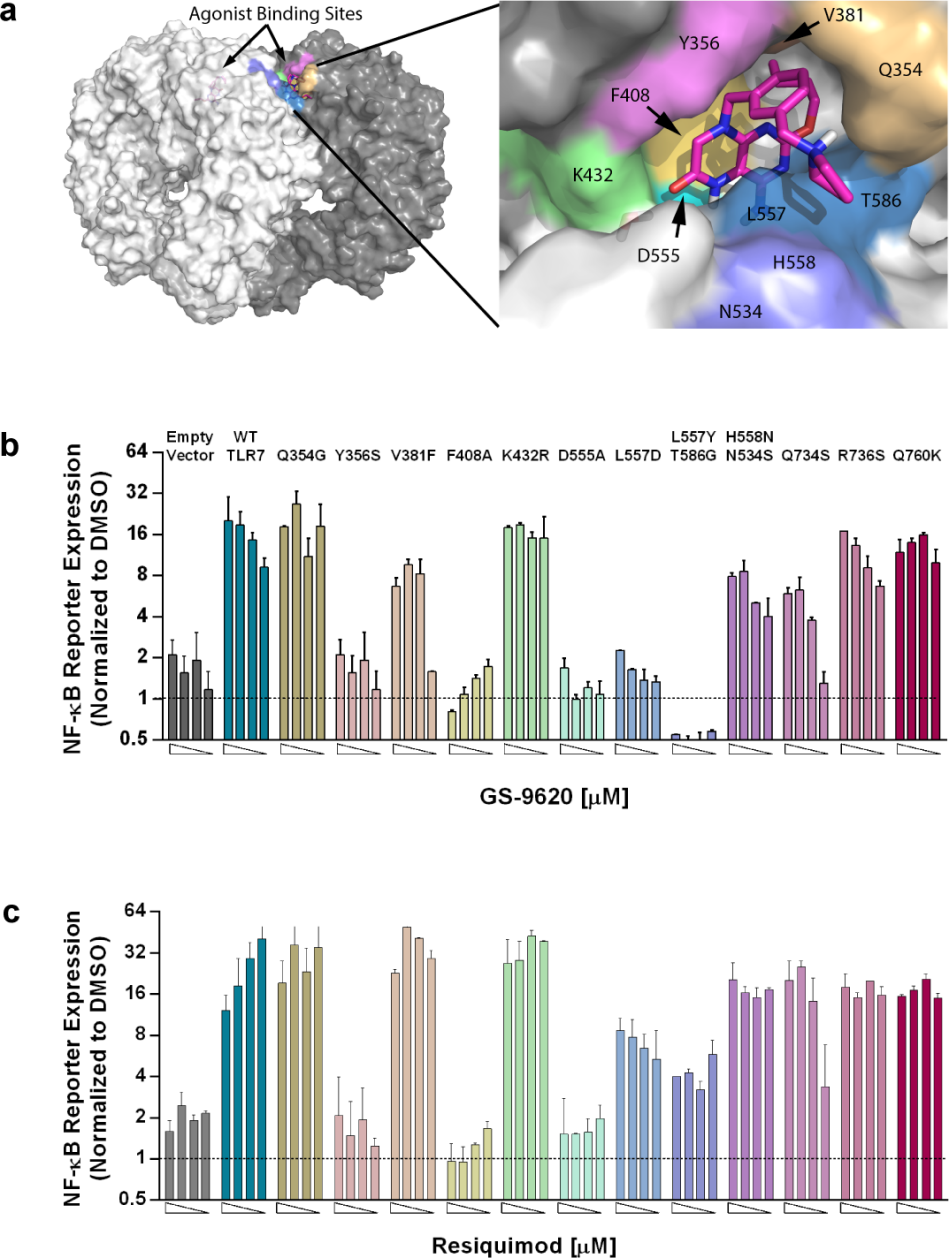
Structure-based mutational analysis identifies residues in TLR7 that are essential for GS-9620 *in vitro* activity. (a) Three dimensional molecular model of TLR7 endo-lysosomal domain (left) and magnified view of GS-9620 docked in TLR7 (right). (b,c) Fold increase in NF-κB-luciferase reporter activity upon GS-9620, resiquimod or DMSO control stimulation in Huh7 cells that were transfected with control vector, wild-type TLR7 or point mutants of TLR7. Four 2-fold dose titrations (left to right) were performed starting at 5μM for GS-9620, or 10 μM of resiquimod. Bar graphs show fold change of reporter activity relative to DMSO control, and error bars shown represent the mean of triplicate assay conditions and SEM. Representative data shown from three independent experiments with similar results. Area under the curve (AUC) calculations were performed to quantify reporter activity observed with titrated compound concentrations for each of the TLR7 variants. With GS-9620 stimulation the variants Y356S, F408A, D555A, L557D, and L557Y/T586G, and with R848 stimulation the variants Y356S, F408A, and D555A show a 4-fold or greater reduction in reporter activity compared to TLR7 WT, as assessed by AUC calculation. Therefore, these variants are viewed as significantly compromised in response to the respective compound.

Next we characterized the molecular interactions required for GS-9620-dependent activation of TLR7. A model of GS-9620 bound to TLR7 has previously been published [27]. The assumed agonist binding pocket is consistent with the pocket identified in crystal structures of TLR8 with resiquimod and other agonists. The pocket is largely conserved between TLR7 and TLR8, but several residues were identified which differ and may be responsible for the TLR7 selectivity of GS-9620. To better understand this selectivity, individual TLR7 residues were mutated to the corresponding amino acid residues in TLR8 (Q354G, K432R, L557D, and N534S/H558N) (S4A Fig). Similarly, residues that were conserved between TLR7 and TLR8, but not TLR9, were mutated to the corresponding amino acid in TLR9 (Y356S, V381F, and L557Y/T586G) (S4A Fig). Key residues conserved between TLR7, TLR8 and TLR9 were mutated to alanine (D555A, F408A). All mutants were assessed for activity in response to stimulation with both GS-9620 and resiquimod (Fig 2B and 2C). Substitutions in residue D555 located in LRR17 affected stimulation by both GS-9620 and resiquimod. Introducing an alanine at the D555 position reduced compound induced reporter activation to baseline (Fig 2B and 2C). This finding is in agreement with previously published studies using other TLR7 agonist compounds [39]. Given that the pKa of GS-9620 is 8.0, in the acidic environment of the lysosome, the pteridinone core of GS-9620 is likely to be protonated and modeling predicts it to form a salt bridge with D555. Modeling also predicts the aromatic ring of F408 neatly forms a parallel stacking interaction with the GS-9620 core (Fig 2A). Mutation of this residue abolished GS-9620- and resiquimod-dependent stimulation. Similarly, Y356 is predicted to form a stabilizing T-like stacking interaction with the GS-9620 core, and mutation to serine abolished GS-9620 and resiquimod-dependent stimulation as well. This latter observation is consistent with the selectivity of these compounds with respect to TLR9.

Mutations of residue L557 resulted in a complete loss of GS-9620-dependent reporter activation but not resiquimod dependent activity. L557 appears to provide the key differentiation between GS-9620 and resiquimod and is likely responsible for the selectivity of GS-9620 toward TLR7 over TLR8. Modeling predicts that the benzyl group in GS-9620 lies over the L557 residue, creating a hydrophobic enclosure (Fig 2A). This interaction is unfavorable when the residue is replaced by an aspartic acid, as found in TLR8. Resiquimod, which does not enclose residue L557, is thus less sensitive to mutation of this residue. Residue V381 contributes to a hydrophobic pocket in TLR7 that the butoxy tail of GS-9620 or ethoxymethyl tail of resiquimod is predicted to occupy. However, substituting the V381 to phenylalanine (V381F) resulted in only a partial loss of activity for GS-9620, but not resiquimod. Neither residues K432 nor Q354 seem to contribute significantly to the binding pocket of GS-9620 or resiquimod, and do not provide additional selectivity as was previously hypothesized [27]. As expected, mutating residues outside of the binding pocket (R736, Q760) did not result in the loss of GS-9620-induced reporter expression, suggesting that the loss of reporter activation through single point mutation analysis was specific to the putative binding site of GS-9620 on TLR7. Importantly, expression of all TLR7 constructs was confirmed using immunoblot analysis suggesting the loss of reporter activity was not due to lack of expression of the TLR7 loss-of-function mutations (S4B Fig). Together these data suggest that residues Y356, V381, and F408 (located in LRR11, 12 and 13, respectively) from one protomer of TLR7 and residues D555, L557 (located in LRR17) from the second protomer together make up the binding pocket of GS-9620. Consequently, two binding pockets for GS-9620 are likely to exist for a single TLR7 dimer. However, our assay system does not permit insights into the stoichiometry of GS-9620 binding to TLR7.

To test if the glycosylation moiety is important to GS-9620 selectivity, residues that are potentially glycosylated in TLR7 and TLR8 were mutated. Residue N354, which is predicted to be glycosylated in TLR7, was mutated to a serine (N354S) and residue H558 (N546 in TLR8) to an asparagine (H558N). Expression of the H558N/N534S double mutant showed partial loss of GS-9620-dependent activation suggesting that glycosylation might have some role in providing TLR7 selectivity to GS-9620. As a control, the NF-κB reporter activity was confirmed for all investigated TLR7 mutants using surrogate stimulation by IL-1β (S4C Fig), because IL-1β was shown to activate the NF-κB pathway via the MyD88 signaling cascade. Irrespective of the TLR7 mutant transfected, Huh7 cells responded to stimulation with IL-1β. This indicates that the various TLR7 mutants did not interfere with the induction of NF-κB-dependent luciferase reporter.

### Validated non-synonymous Single Nucleotide Polymorphisms do not impact GS-9620 dependent TLR7 activation

**Fig 3:**
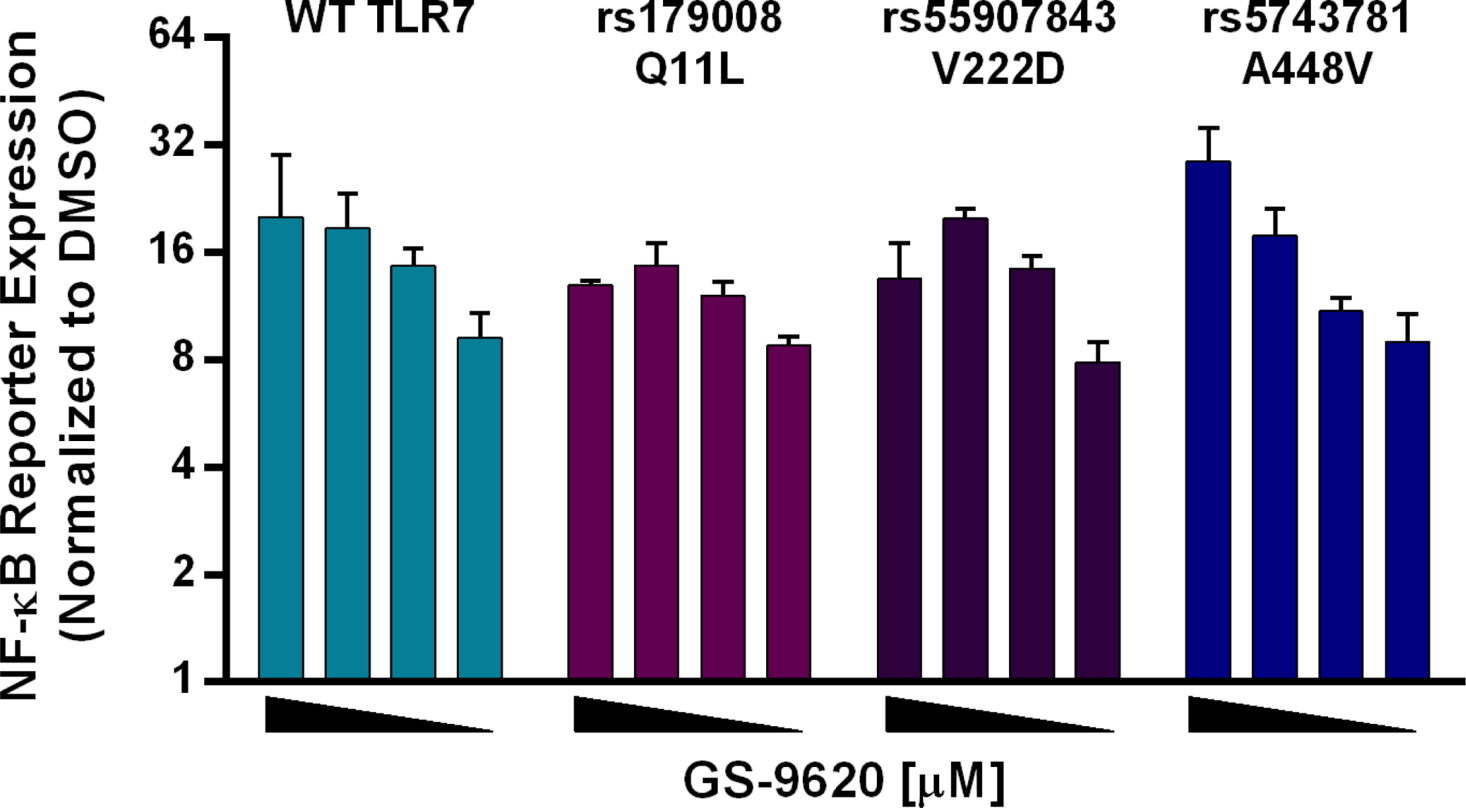
Amino acid changes due to described single nucleotide polymorphisms (SNPs) do not impact GS-9620-dependent TLR7 activation. Fold increase in NF-kB-luciferase reporter activity upon GS-9620 stimulation in Huh7 cells that were transfected with TLR7 or SNPs rs179008 (Q11L), rs55907843 (V222D), or rs5743781 (A448V). Four 2-fold dose titration curves were performed starting at 5µM for GS-9620 (left to right). Bar graphs show fold change in reporter activity relative to DMSO, and error bars represent the mean of triplicate assay conditions and SEM. AUC calculations were performed as discussed for Fig 2, above. By the same AUC criteria none of the three assessed TLR7 SNP variants elicited a significantly altered response, relative to TLR7 WT, after GS-9620 stimulation. Representative data are shown from three independent experiments with similar results.

Although population based sequencing efforts have identified lesser sequence diversity in human *TLR7*, compared to other TLR genes, several non-synonymous single nucleotide polymorphisms (SNPs) for *TLR7* have been reported in the dbSNP [40]. Some non-synonymous SNPs, such as rs179008 (Q11L), rs55907843 (V222D) and rs5743781 (A448V) were observed at very low population frequencies and have been validated by independent submissions. These naturally occurring non-synonymous SNPs were tested in our reporter assay system. None of the tested SNPs rs179008 (Q11L), rs55907843 (V222D) or rs5743781 (A448V) had a detectable impact on GS-9620-dependent TLR7 reporter activation (Fig 3). These findings suggest that non-synonymous SNPs are unlikely to interfere with GS-9620-dependent TLR7 activation and are consistent with the GS-9620/TLR7 interaction surface inferred from the mutation analysis data presented above.

### TLR7 form constitutive dimers

**Fig 4:**
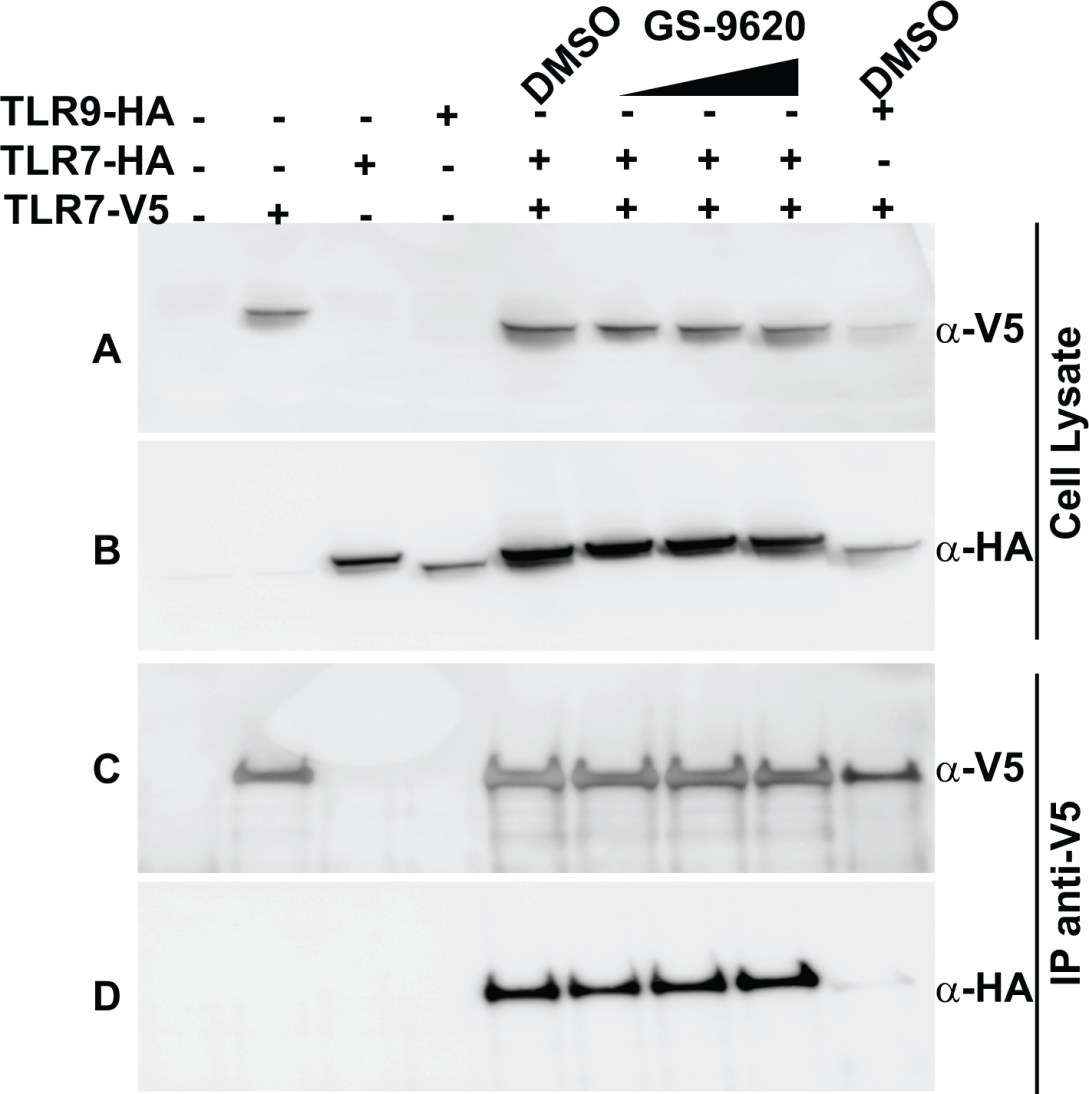
TLR7 dimers exist independent of GS-9620 binding. Immunoblot analysis of whole cell lysates of Huh7 cells transfected with V5-tagged TLR7 (TLR7-V5) or HA-tagged TLR7 (TLR7-HA) or HA-tagged TLR9 (TLR9-HA) untreated or treated with increasing amounts of GS-9620 (0.1μM, 1μM and 5μM) for 1 hour before preparation of cell lysates and immunoprecipitation (IP) analysis. Lysates were immunoprecipitated with anti-V5 agarose (panel C,D) and immunoblot probed with anti-V5 mAb (panel A,C) or anti-HA mAb (panel B,D). Total cell lysates used in panels A and B were probed with anti-V5 mAb and anti-HA mAb respectively, to control for protein expression and loading.

Dimerization of endo-lysosomal TLRs is an important component in receptor function. In contrast to TLR3, TLR8 and TLR9 were shown to be exist as preformed dimers, independent of ligand binding. Ligand-induced conformational changes in the TIR domains have been reported for TLR8 and TLR9 [9, 10]. To assess the oligomeric state of TLR7, co-immunoprecipitation experiments were performed using Huh7 cells that were transfected with two differentially epitope-tagged versions of TLR7 (TLR7-V5 and TLR7-HA). Immunoprecipitation using anti-V5 antibody also precipitated TLR7-HA, but not TLR9-HA (Fig 4). This co-immunoprecipitation of TLR7-HA occurred both in the presence or absence of GS-9620. Furthermore, increasing concentrations of GS-9620 did not increase the amount of co-immunoprecipitated TLR7-HA. Collectively, these data suggest that TLR7 can exist as pre-formed dimers in the absence of ligand binding, although we cannot rule out the possibility that over-expression of TLR7 in this system results in non-physiologic oligomeric states. Poor specificity of available anti-TLR7 antibodies precluded the possibility of extending these findings using endogenous TLR7. The finding that the TLR7 loss-of-function mutants F408A and D55A do not result in loss of receptor dimerization, suggests that the identified loss of function mutations do not result in gross perturbations of the TLR7 structure (S5 Fig). Further evidence for the concept of dimeric TLR7 protein was obtained by analysis of full-length recombinant (rec) TLR7 expressed and purified from insect cells. Size exclusion chromatography analysis detected the recTLR7 protein at a retention time suggestive of a dimeric state (S6 Fig). By extrapolation from data published for TLR8 and TLR9, GS-9620 binding to a pre-formed TLR7 dimer likely results in a change in conformation that brings the cytoplasmic TIR domains in close proximity, stabilizing an active conformation which in turn recruits the adaptor molecule MyD88 to initiate signal transduction [9, 10].

### GS-9620 induces the phosphorylation of NF-κB and Akt in plasmacytoid dendritic cells

**Fig 5:**
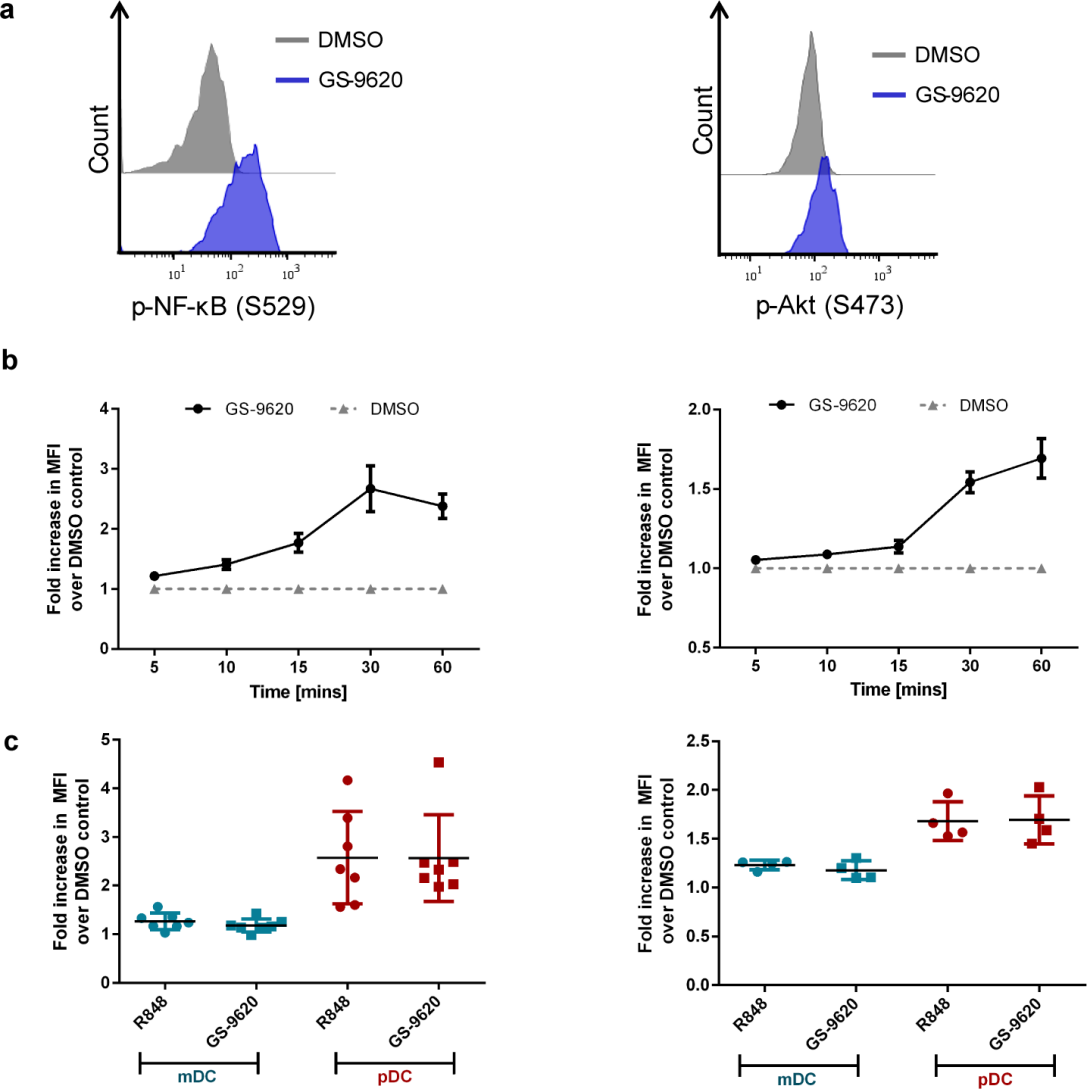
GS-9620 induces phosphorylation of NF-κB and Akt in pDCs. PBMC isolated from healthy donors were cultured with GS-9620, resiquimod, or DMSO control. Phosphorylation of NF-κB and Akt was assessed in gated pDC and mDC subsets. (a) Flow cytometric histogram of p-NF-κB (using a phosho NF-κB (S529) specific antibody) following 30min stimulation (left panel) or p-Akt (using a phospho Akt (S473) specific antibody) following 60min stimulation (right panel) with 1μM GS-9620 (blue histogram) or DMSO control (grey histogram). (b) Fold increase in mean fluorescence intensity (MFI) of pDCs for pNF-κB (left panel) or p-Akt (right panel) after stimulation with 1μM GS-9620 normalized to DMSO control treatment. Statistically significant differences relative to DMSO control (p<0.05) are observed with GS-9620 stimulation for all assessed time points (p-NF-κB), and for 10, 15, 30, and 60min time points (p-Akt). (c) Fold increase in MFI upon stimulation with 1μM GS-9620 or resiquimod (R848) normalized to DMSO control treatment in mDCs or pDCs. As expected, phospho-responses to GS-9620 and R848 for both readouts, p-NF-κB and p-Akt, were significantly stronger in pDC compared to donor-matched mDC (p<0.01 for all comparisons). Graphs show data obtained from 6 (p-NF-κB) and 4 (p-Akt) independent healthy donors with mean ±SEM (bars).

pDCs are important to the innate antiviral immune response by producing type I IFN and also play a major role in adaptive immune responses as antigen presenting cells. TLR activation in pDCs culminates in secretion of cytokines and chemokines as well as in the upregulation of co-stimulatory molecules [23] [41]. The molecular pathways mediating TLR-dependent activation of pDCs converge on several signaling switches including, NF-κB and PI(3)K-Akt [19] [20] [13] [42]. We sought to understand the downstream signaling events elicited by GS-9620 in human pDCs. PBMCs from healthy volunteers were stimulated using either GS-9620, resiquimod or DMSO control over a range of time points and phosphorylation of NF-κB and Akt were measured using phospho-specific antibodies by flow cytometry. GS-9620 induced phosphorylation of NF-κB p65 (pS529) within 10 minutes, peaking at 30 minutes post-stimulation. In contrast, phosphorylation of Akt (pS473) had slightly slower kinetics, peaking at 60 minutes (Fig 5B). Over 50% of pDCs were positive for phopho-NF-κB after 30 minutes while roughly 20–30% of pDCs were positive for phospho-Akt at 60 minutes (S7 Fig). Both phosphorylation events induced by GS-9620 were observed specifically in pDCs and not in mDCs (Fig 5C) and were dose-dependent (S8 Fig). These results are consistent with the observation that pDCs selectively express TLR7, which is not detected in mDCs [18, 43]. Together, these data indicate the GS-9620 activates both NF-κB and Akt, key signaling factors that mediate several downstream responses in pDCs.

## Conclusions

The present work characterizes the molecular interactions and mechanism-of-action of the small molecule TLR7 agonist GS-9620. The physiochemical properties of GS-9620 allow the small molecule to rapidly and selectively enter endo-lysosomal compartments where it binds to TLR7 in a pH-sensitive manner. We show evidence to suggest that TLR7 likely stabilizes itself through adopting a homo-dimer conformation independent of ligand, similar to TLR8 and TLR9. Using results from structure guided mutational studies, we propose that amino acids D555 and D557 in LRR17 region from one monomer and amino acids Y356 in LRR11 domain and F408 in LRR13 domain from the second monomer together form a binding pocket for GS-9620. We report that the described single-nucleotide polymorphisms (SNPs) in the coding region of TLR7 do not impact activation by GS-9620. Studies using healthy human donor PBMCs stimulated with GS-9620 in conjunction with genotypic analysis of the *TLR7* locus for polymorphisms are currently being conducted. Finally, flow cytometric analysis of dendritic cell subsets demonstrate that the phosphorylation of NF-κB and Akt constitute early signaling events in pDCs in response to activation of TLR7. Together, the data reported here enhance our understanding of TLR7 activation by the small molecule agonist GS-9620.

## Supporting Information

S1 Fig Sub-cellular distribution of major organelle markers obtained by percoll gradient centrifugation. Organelle makers include ER marker (Cytochrome *c* reductase (NADPH)), mitochondrial marker (Mitochondrial succinate dehydrogenase), and lysosomal marker (β-hexosaminidase) (Figure A). Immunoblot of early endosomal marker Rab5 (Figure B).
(TIF)

S2 Fig Vesicular pH measurement. Emission of FITC-dextran (at 530nM) in buffer with increasing pH, upon excitation spectral scan (400nM to 515nM) (Figure A). Change in endo-lysosomal pH upon bafilomycin A1 treatment as measured by change in FITC emission at 530nM (Figure B).
(TIF)

S3 Fig Specificity of GS-9620 using Huh-7 cells expressing TLR7, and the effect of bafilomycin A1 on GS-9620-dependent reporter activation. Huh-7 cells were transfected with human TLR7 and TLR9 (Figure A, Figure B). Fold increase in NF-κB-driven luciferase reporter activity upon stimulation with GS-9620 (Figure A) or ODN 2216 (Figure B) was assessed to demonstrate specificity ofGS-9620 for TLR7 in this system. Huh-7 cells were transfected with human TLR7 only (Figure C, Figure D). Cells were stimulated with GS-9620 in the presence of bafilomycin A1 (BAF) or PBS (mock) to demonstrate effect of endo-lysosomal pH on GS-9620 activity (Figure C). As a control the same cells were stimulated with IL-1β to show that BAF did not interfere with the MyD88-dependent signaling in this setting (Figure D). Five point 2-fold dose titration curves were performed starting at 5µM for GS-9620, 15µM for ODN 2216, 12.5ng/ml IL-1β (left to right). Data is the mean of triplicates ±SEM (bars). Representative data are shown from 3 independent experiments with similar results.
(TIF)

S4 Fig Structure based mutational analysis of TLR7. Table listing corresponding amino acids in human TLR7, TLR8 and TLR9, as determined by structure homology modeling-guided sequence alignment, and the mutations tested in the study (Figure A). Fold increase in NF-κB-driven luciferase reporter activity upon IL-1β stimulation in Huh7 cells that were transfected with control vector (pUNO), GFP, WT TLR7, point mutations of TLR7, or SNPs of TLR7. Reporter activity was normalized to DMSO control. Four 2-fold dose titration curves were performed starting at 12.5ng/ml IL-1β (left to right). Data is the mean of triplicates ±SEM (bars). Representative data are shown from 3 independent experiments with similar results (Figure B). Immunoblot analysis of whole cell lysates confirming the expression of TLR7 and respective point mutants and SNP mutations using a polyclonal antibody against human TLR7 (top row) or anti-GAPDH Ab as loading control (bottom row) (Figure C).
(TIF)

S5 Fig TLR7 dimerization is not perturbed by tested point mutations. Immunoblot analysis of whole cell lysates of Huh7 cells transfected with V5-tagged point mutants of TLR7 (F408A-V5) or (D555A-V5) or corresponding HA-tagged point mutants of TLR7 (F408A-HA) or (D555A-HA). Lysates were immunoprecipitated with anti-V5 agarose (panels C,D) and after separation of immunoprecipitated proteins by SDS-PAGE were probed with anti-V5 mAb (panels A,C) or anti-HA mAb (panels B,D). Total cell lysates in panels A and B and probed with anti-V5 mAb and anti-HA mAb respectively to assess protein expression and control for protein loading.
(TIF)

S6 Fig Size exclusion chromatography analysis of recombinant human TLR7 suggests a dimeric protein species. Coomassie staining of purified TLR7 protein, as obtained from the 15 ml elution fraction, analyzed by SDS-PAGE (Figure A). Size exclusion chromatogram of purified recombinant full length TLR7 (black curve) and calibration standards (grey) including (670kDa, Thyroglobulin), (158kDa, Gamma-globulin), (44kDa, Ovalbumin), (17kDa; Myoglobin) and (1.35 kDa, VitaminB12) (Figure B).
(TIF)

S7 Fig Frequency of p-NF-κB^+^ and p-Akt^+^ pDC following GS-9620 stimulation. Gating strategy for defining of mDC and pDC subsets from a representative donor. Lineage cocktail contained CD3, CD14, CD16 CD19, CD20, and CD56 (Figure A). Frequency of p-NF-κB^+^ pDCs (Figure B) or p-Akt^+^ pDCs (Figure C) over time following stimulation with 1μM of GS-9620 or DMSO control. Statistically significant differences relative to DMSO control (p<0.05) are observed with GS-9620 stimulation for 10, 15, 30, and 60min time points (p-NF-κB), and for all assessed time points (p-Akt). Data is mean ±SEM (bars) representing 6 (p-NF-κB) and 4 (p-Akt) different healthy donors.
(TIF)

S8 Fig Dose dependent induction of NF-κB and Akt phosphorylation by GS-9620. Dose dependent increase in p-NF-κB (Figure A) or p-Akt (Figure B) activation upon stimulation with GS-9620 compared to DMSO control following 30 or 60 minute stimulation, respectively. A composite score “MFI x frequency” is used to capture the magnitude of the phospho-response induced by GS-9620. This score combines the parameters of cell frequency and MFI for the phospho-positive flow cytometry events. Statistically significant differences relative to DMSO control (p<0.05) are observed at GS-9620 concentrations equal or greater than 0.125 µM (p-NF-κB) and equal or greater than 0.5 µM (p-Akt). Data is mean ±SEM (bars) representing 6 (p-NF-κB) and 4 (p-Akt) different healthy donors.
(TIF)
