# Supplementary figures and images for: Molecular Determinants of GS-9620-Dependent TLR7 Activation
Source: PLoS One. 2016 Jan 19;11(1):e0146835. doi: 10.1371/journal.pone.0146835 (PMC4718629; doi:10.1371/journal.pone.0146835)

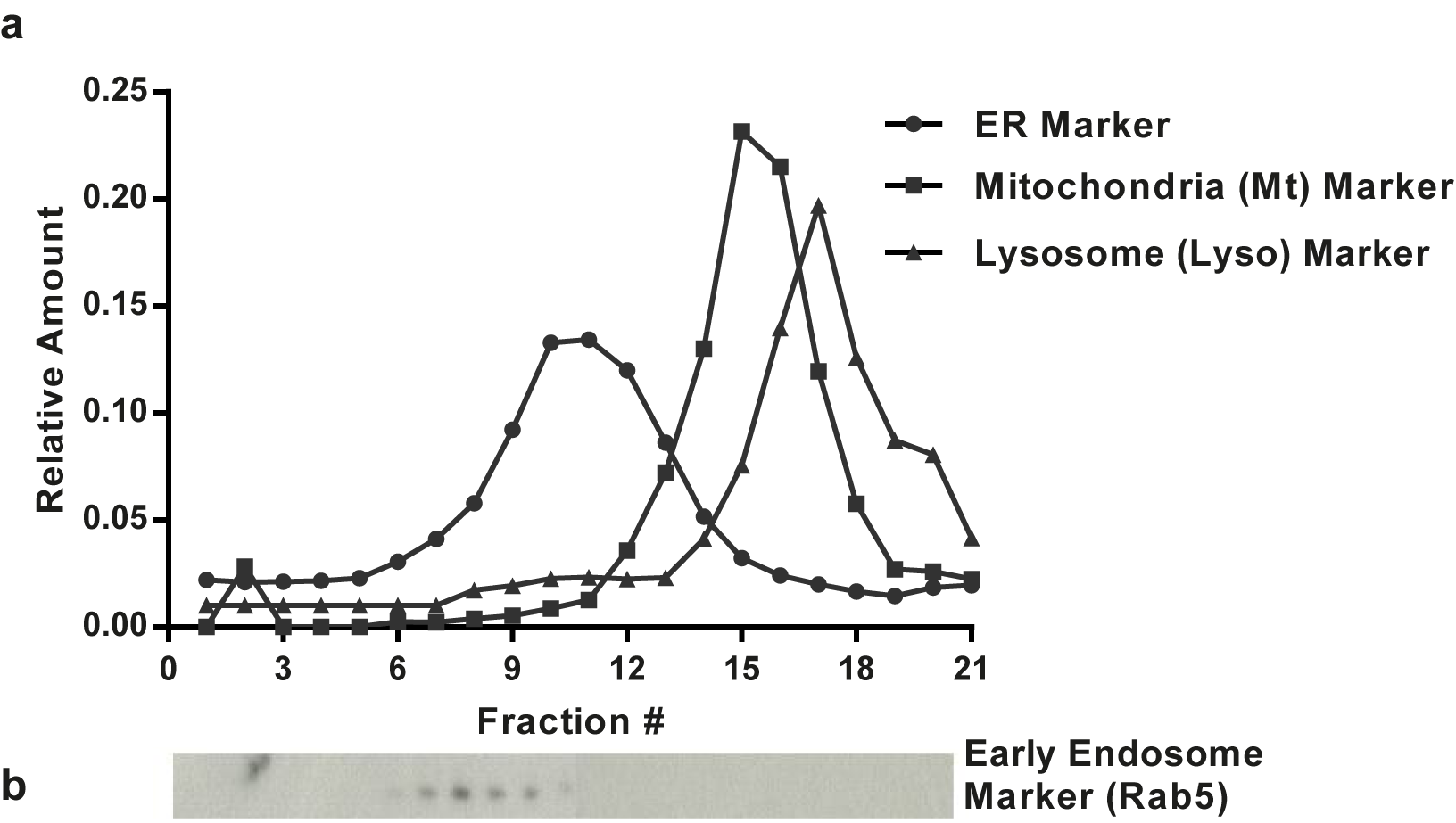

Supplement: S1 Fig — Organelle makers include ER marker (Cytochrome c reductase (NADPH)), mitochondrial marker (Mitochondrial succinate dehydrogenase), and lysosomal marker (β-hexosaminidase) (Figure A). Immunoblot of early endosomal marker Rab5 (Figure B). (TIF) [file pone.0146835.s001.tif]

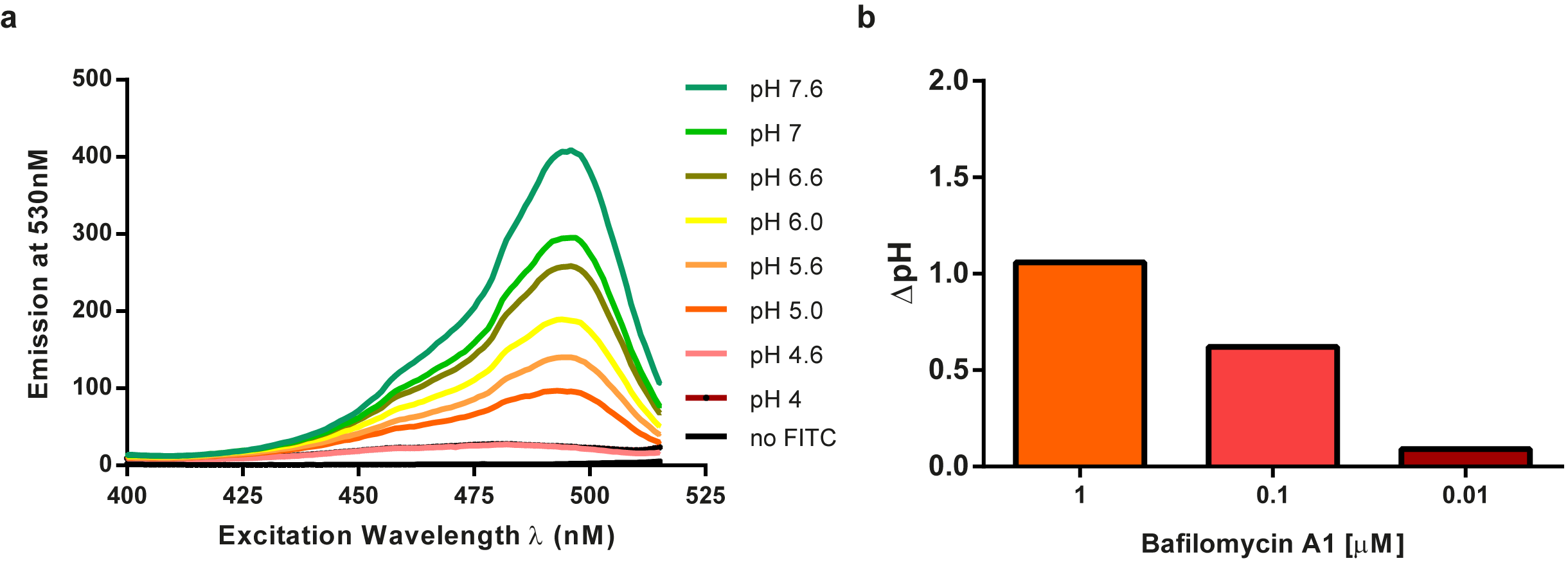

Supplement: S2 Fig — Emission of FITC-dextran (at 530nM) in buffer with increasing pH, upon excitation spectral scan (400nM to 515nM) (Figure A). Change in endo-lysosomal pH upon bafilomycin A1 treatment as measured by change in FITC emission at 530nM (Figure B). (TIF) [file pone.0146835.s002.tif]

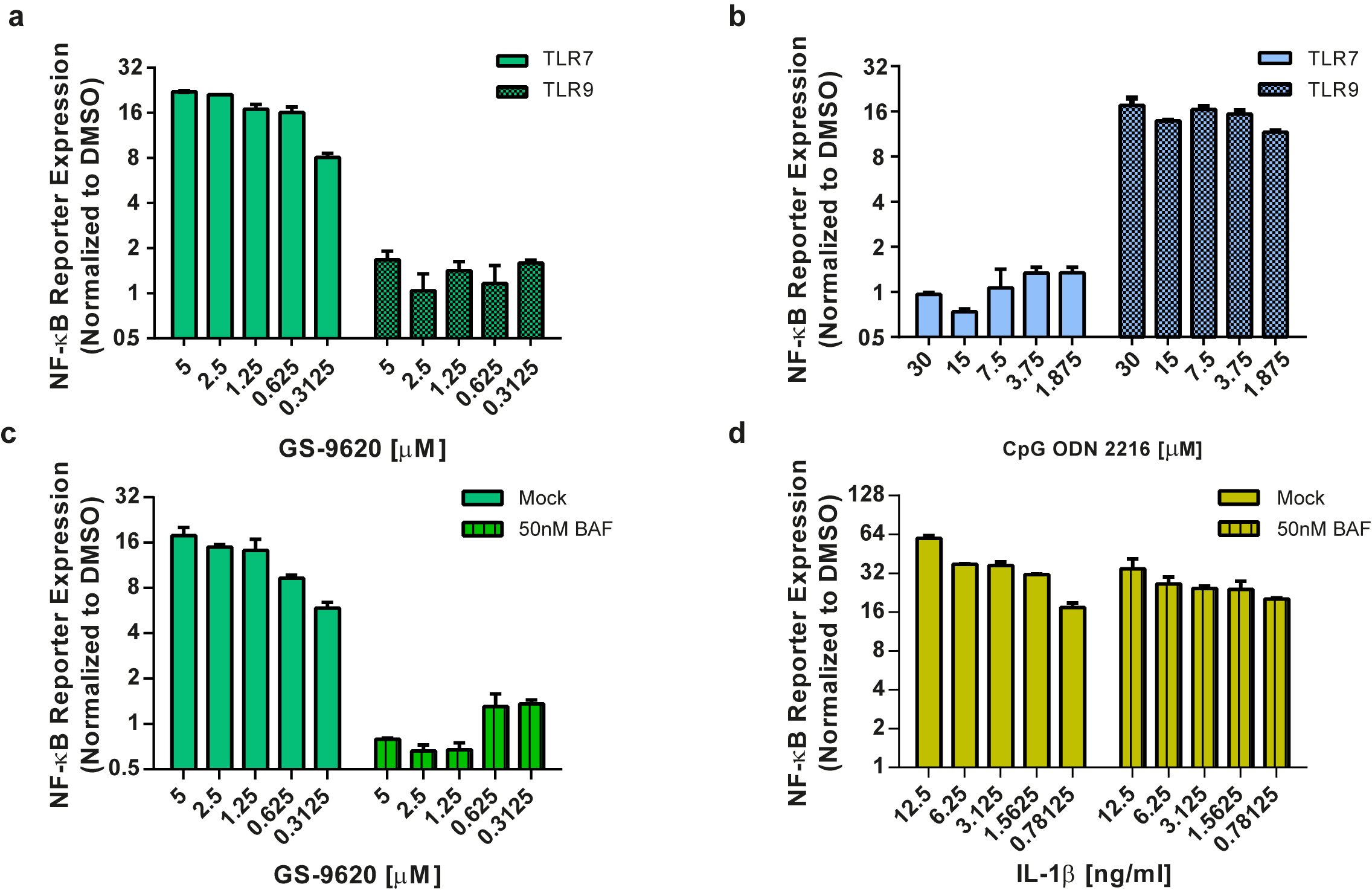

Supplement: S3 Fig — Huh-7 cells were transfected with human TLR7 and TLR9 (Figure A, Figure B). Fold increase in NF-κB-driven luciferase reporter activity upon stimulation with GS-9620 (Figure A) or ODN 2216 (Figure B) was assessed to demonstrate specificity ofGS-9620 for TLR7 in this system. Huh-7 cells were transfected with human TLR7 only (Figure C, Figure D). Cells were stimulated with GS-9620 in the presence of bafilomycin A1 (BAF) or PBS (mock) to demonstrate effect of endo-lysosomal pH on GS-9620 activity (Figure C). As a control the same cells were stimulated with IL-1β to show that BAF did not interfere with the MyD88-dependent signaling in this setting (Figure D). Five point 2-fold dose titration curves were performed starting at 5µM for GS-9620, 15µM for ODN 2216, 12.5ng/ml IL-1β (left to right). Data is the mean of triplicates ±SEM (bars). Representative data are shown from 3 independent experiments with similar results. (TIF) [file pone.0146835.s003.tif]

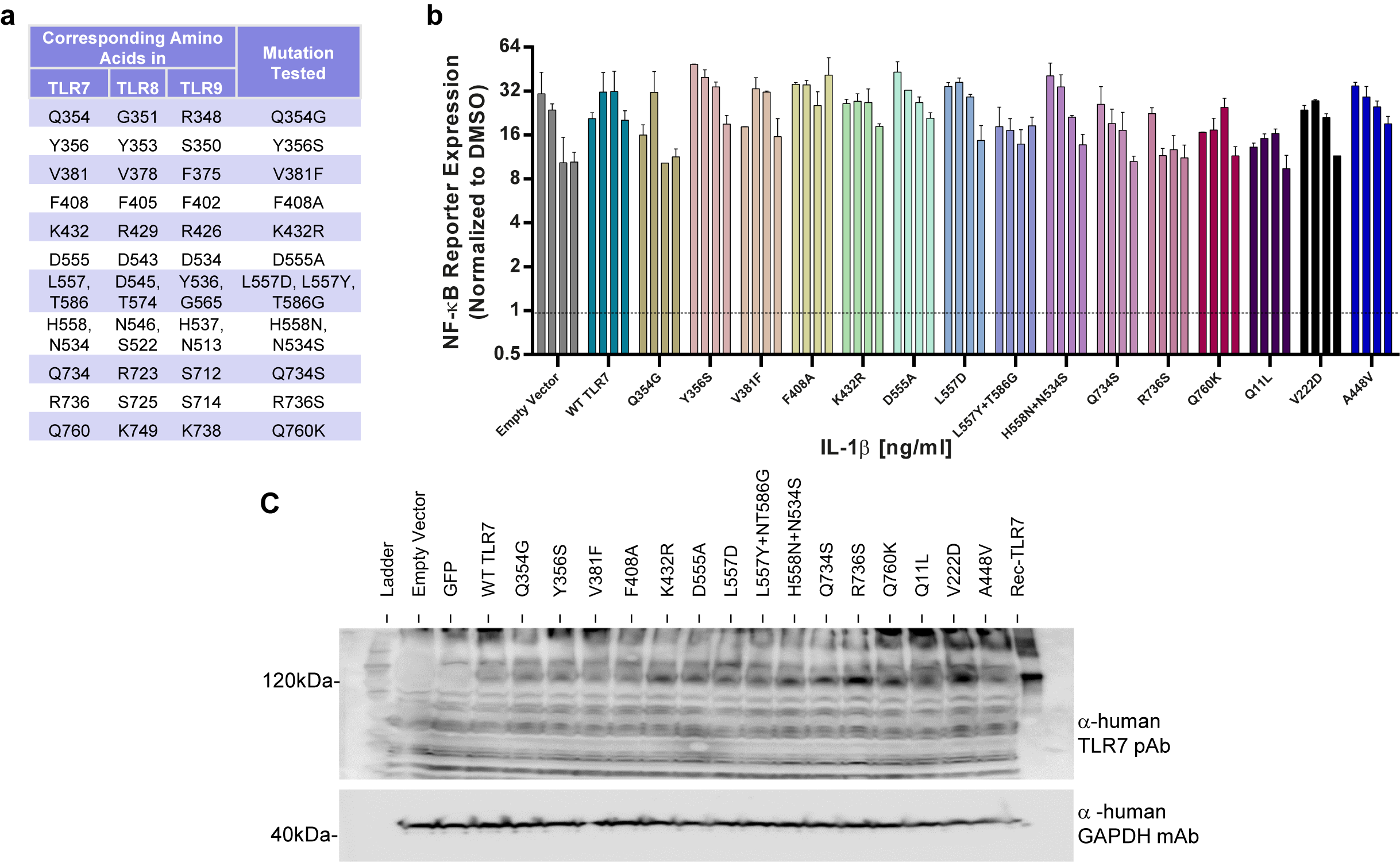

Supplement: S4 Fig — Table listing corresponding amino acids in human TLR7, TLR8 and TLR9, as determined by structure homology modeling-guided sequence alignment, and the mutations tested in the study (Figure A). Fold increase in NF-κB-driven luciferase reporter activity upon IL-1β stimulation in Huh7 cells that were transfected with control vector (pUNO), GFP, WT TLR7, point mutations of TLR7, or SNPs of TLR7. Reporter activity was normalized to DMSO control. Four 2-fold dose titration curves were performed starting at 12.5ng/ml IL-1β (left to right). Data is the mean of triplicates ±SEM (bars). Representative data are shown from 3 independent experiments with similar results (Figure B). Immunoblot analysis of whole cell lysates confirming the expression of TLR7 and respective point mutants and SNP mutations using a polyclonal antibody against human TLR7 (top row) or anti-GAPDH Ab as loading control (bottom row) (Figure C). (TIF) [file pone.0146835.s004.tif]

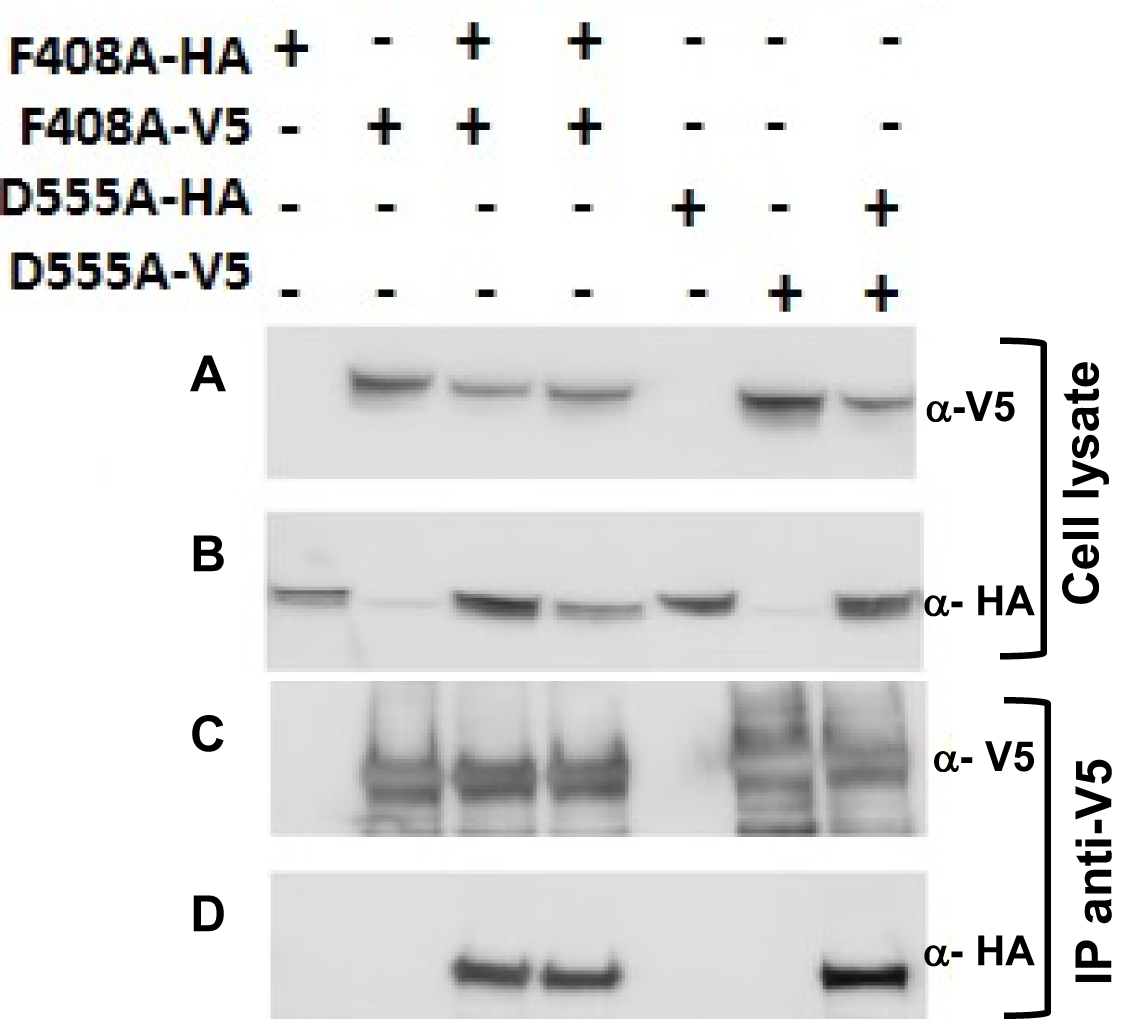

Supplement: S5 Fig — Immunoblot analysis of whole cell lysates of Huh7 cells transfected with V5-tagged point mutants of TLR7 (F408A-V5) or (D555A-V5) or corresponding HA-tagged point mutants of TLR7 (F408A-HA) or (D555A-HA). Lysates were immunoprecipitated with anti-V5 agarose (panels C,D) and after separation of immunoprecipitated proteins by SDS-PAGE were probed with anti-V5 mAb (panels A,C) or anti-HA mAb (panels B,D). Total cell lysates in panels A and B and probed with anti-V5 mAb and anti-HA mAb respectively to assess protein expression and control for protein loading. (TIF) [file pone.0146835.s005.tif]

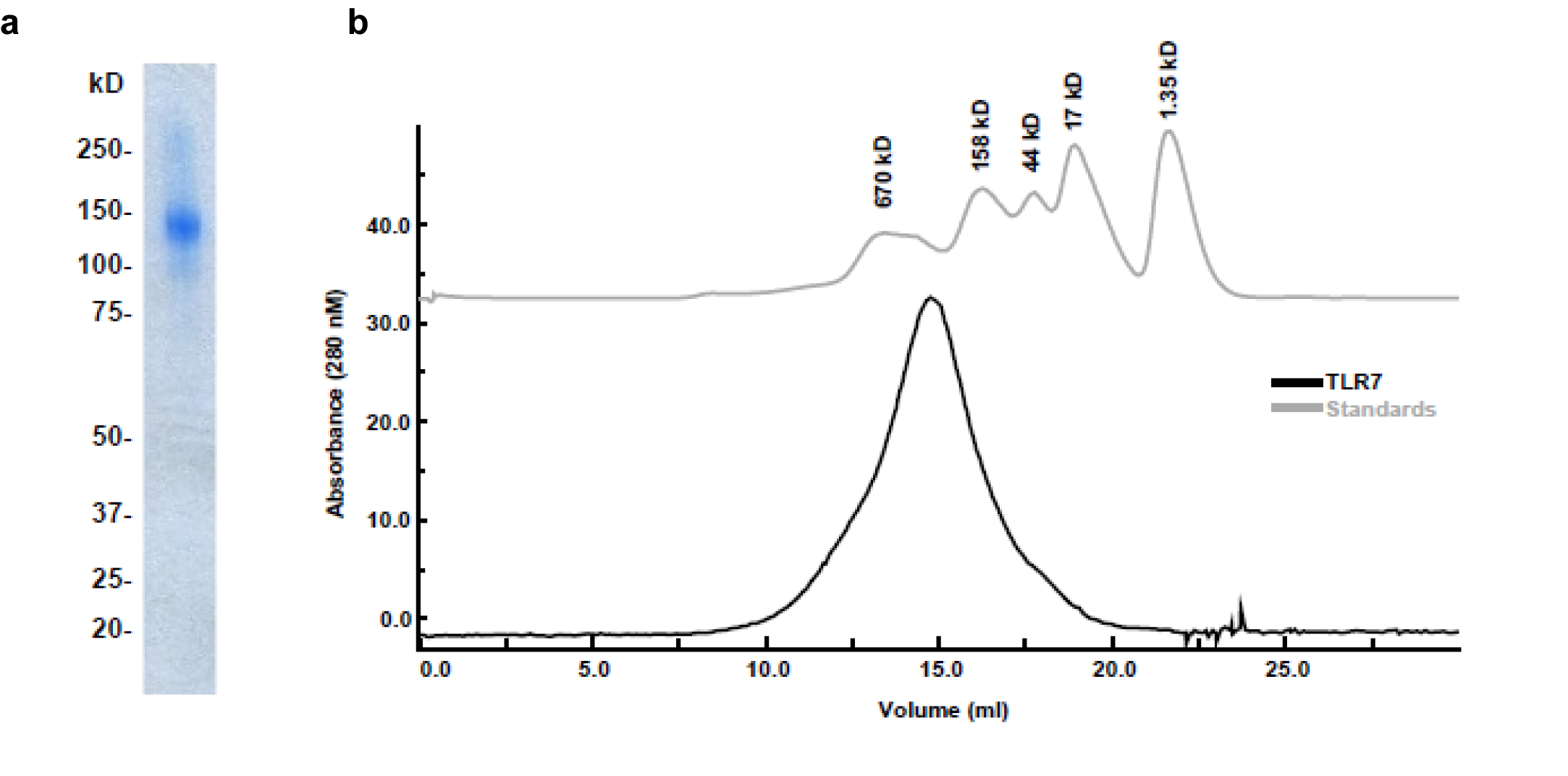

Supplement: S6 Fig — Coomassie staining of purified TLR7 protein, as obtained from the 15 ml elution fraction, analyzed by SDS-PAGE (Figure A). Size exclusion chromatogram of purified recombinant full length TLR7 (black curve) and calibration standards (grey) including (670kDa, Thyroglobulin), (158kDa, Gamma-globulin), (44kDa, Ovalbumin), (17kDa; Myoglobin) and (1.35 kDa, VitaminB12) (Figure B). (TIF) [file pone.0146835.s006.tif]

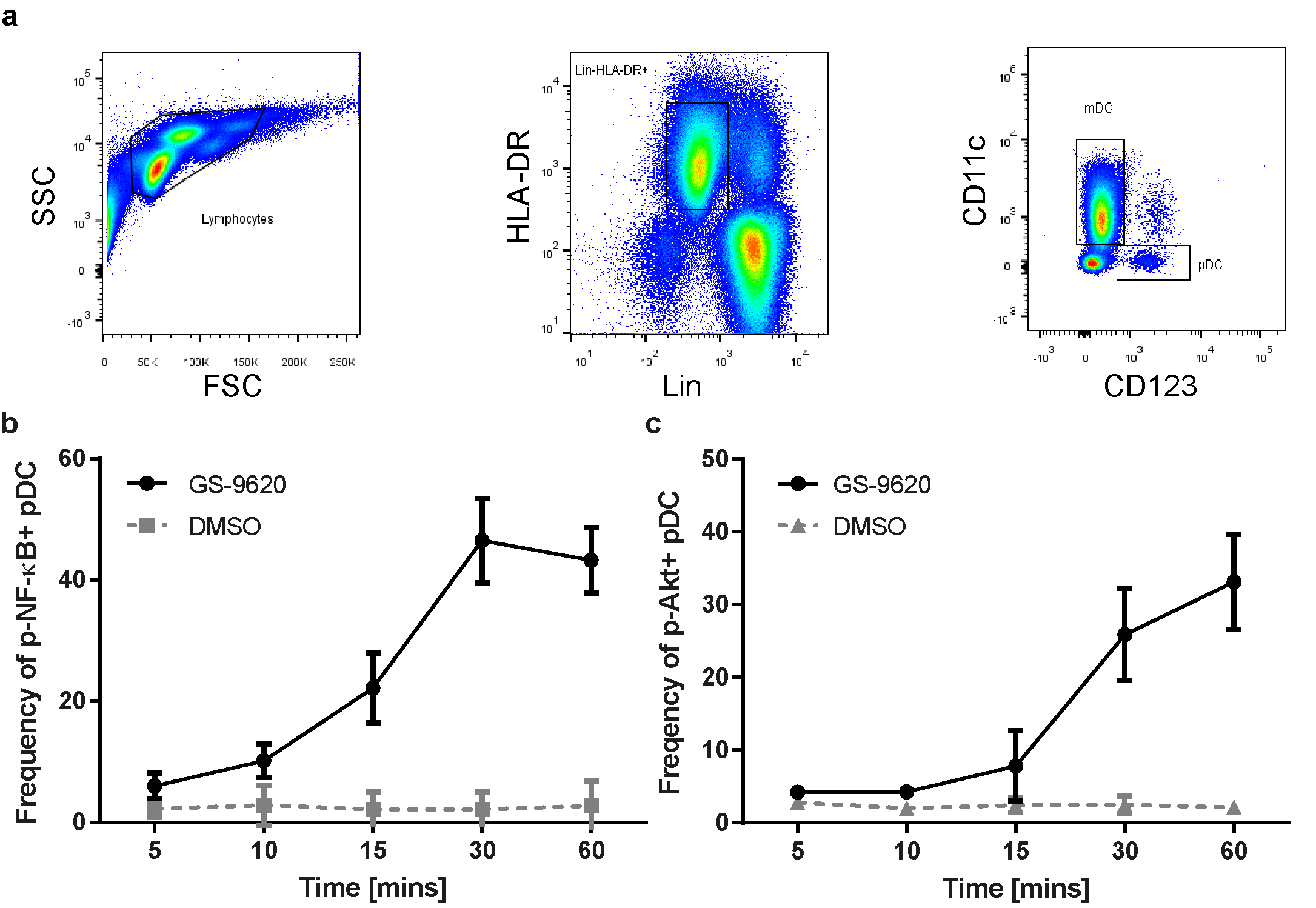

Supplement: S7 Fig — Gating strategy for defining of mDC and pDC subsets from a representative donor. Lineage cocktail contained CD3, CD14, CD16 CD19, CD20, and CD56 (Figure A). Frequency of p-NF-κB+ pDCs (Figure B) or p-Akt+ pDCs (Figure C) over time following stimulation with 1μM of GS-9620 or DMSO control. Statistically significant differences relative to DMSO control (p<0.05) are observed with GS-9620 stimulation for 10, 15, 30, and 60min time points (p-NF-κB), and for all assessed time points (p-Akt). Data is mean ±SEM (bars) representing 6 (p-NF-κB) and 4 (p-Akt) different healthy donors. (TIF) [file pone.0146835.s007.tif]

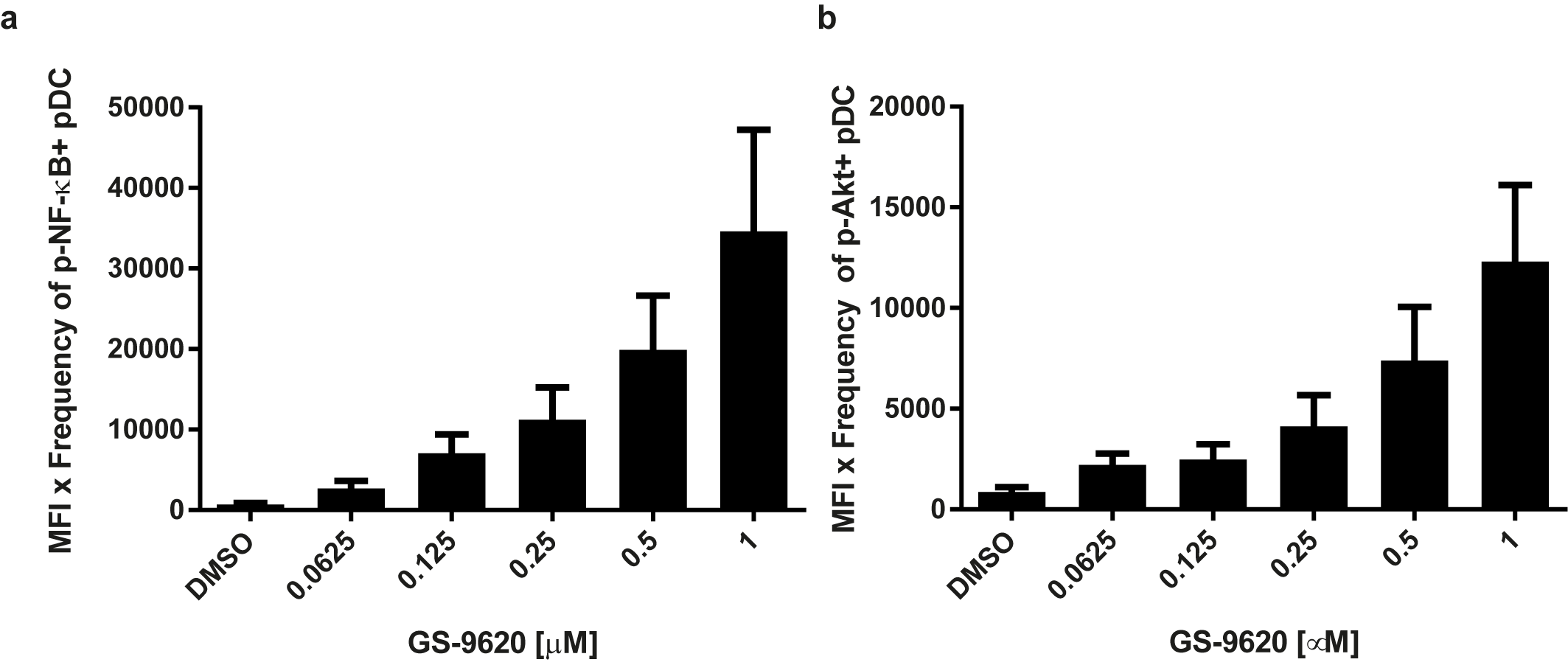

Supplement: S8 Fig — Dose dependent increase in p-NF-κB (Figure A) or p-Akt (Figure B) activation upon stimulation with GS-9620 compared to DMSO control following 30 or 60 minute stimulation, respectively. A composite score “MFI x frequency” is used to capture the magnitude of the phospho-response induced by GS-9620. This score combines the parameters of cell frequency and MFI for the phospho-positive flow cytometry events. Statistically significant differences relative to DMSO control (p<0.05) are observed at GS-9620 concentrations equal or greater than 0.125 µM (p-NF-κB) and equal or greater than 0.5 µM (p-Akt). Data is mean ±SEM (bars) representing 6 (p-NF-κB) and 4 (p-Akt) different healthy donors. (TIF) [file pone.0146835.s008.tif]
